# Supplementary material for: Congruence between noise and plasticity in protein expression
Source: Sci Rep. 2025 Jul 8;15:24529. doi: 10.1038/s41598-025-07594-2 (PMC12238574; doi:10.1038/s41598-025-07594-2)
Supplement: Supplementary file 1 — Supplementary Information 1. [file 41598_2025_7594_MOESM1_ESM.pdf]

Supplementary information for Congruence between noise and plasticity in protein expression

Saburo Tsuru[1,*] and Chikara Furusawa[1,2,3,*]

[1]Universal Biology Institute, Graduate School of Science, The University of Tokyo, 7-3-1 Hongo, Bunkyo-ku, Tokyo 113-0033, Japan
[2]Department of Physics, Graduate School of Science, The University of Tokyo, 7-3-1 Hongo, Bunkyo-ku, Tokyo 113-0033, Japan
[3]Center for Biosystems Dynamics Research (BDR), RIKEN, 6-7-1 Minatojima-minamimachi, Chuo-ku, Kobe 650-0047, Japan

*Corresponding authors:
Saburo Tsuru (tsuru@ubi.s.u-tokyo.ac.jp)
Chikara Furusawa (chikara.furusawa@riken.jp)

I. Supplementary Figures
II. Supplementary References

I. Supplementary Figures

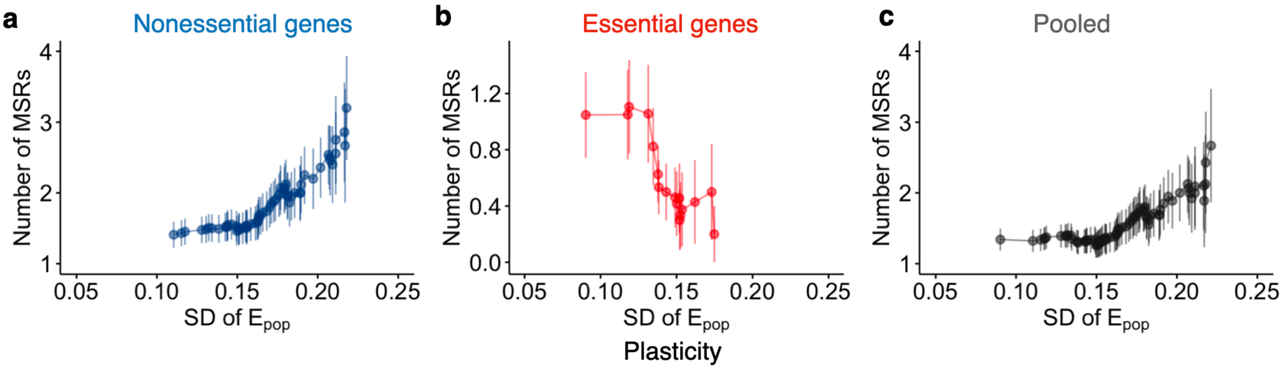

Fig. S1: Relationship between the number of transcriptional regulators and plasticity
Following a previous study (Figure 2 in Wolf *et al*[1]), a cumulative approach was employed to capture the relationship between the number of metabolic sensory regulators (MSRs) and plasticity in protein expression levels. Genes were sorted based on their plasticity levels (x). For each cut-off value on x (horizontal axis), the mean and standard error (vertical axis) of the number of MSRs were calculated for all genes with plasticity levels greater than x. The first cut-off (rightmost point) was set at the plasticity level of the sixth gene in the sorted list to ensure that each data point and error bar correspond to more than four genes for all panels. Mean values and standard errors are shown as the points and error bars, respectively. Nonessential (**a**), essential (**b**), and pooled genes (**c**) were considered separately.

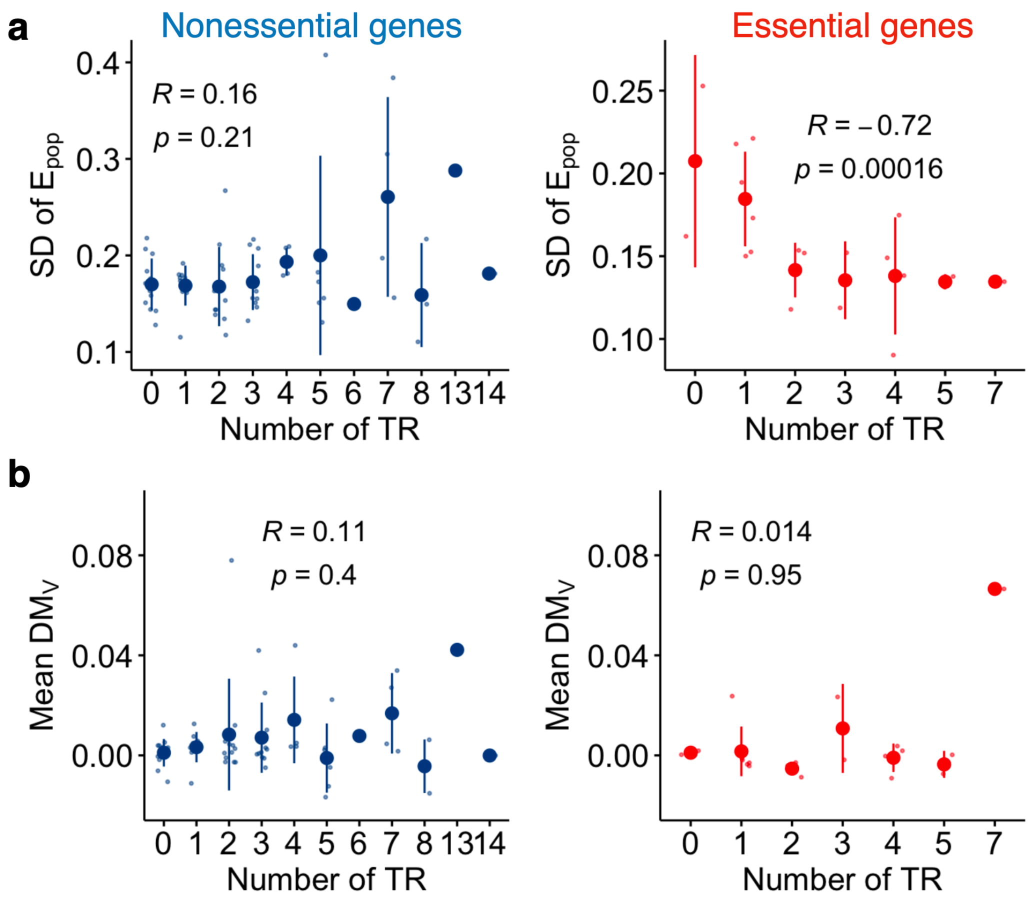

Fig. S2: Relationship between the number of transcriptional regulators, plasticity, and noise
a, Relationship between the number of transcriptional regulators (TR) and plasticity in protein expression levels (SD of E_{pop}). All transcriptional regulators including MSRs and other TRs were considered. **b**, Relationship between the number of TRs and noise in protein expression level (mean DM_V). Means and standard deviations are shown as the large points and the error bars, respectively. Spearman's R and p-value are shown.

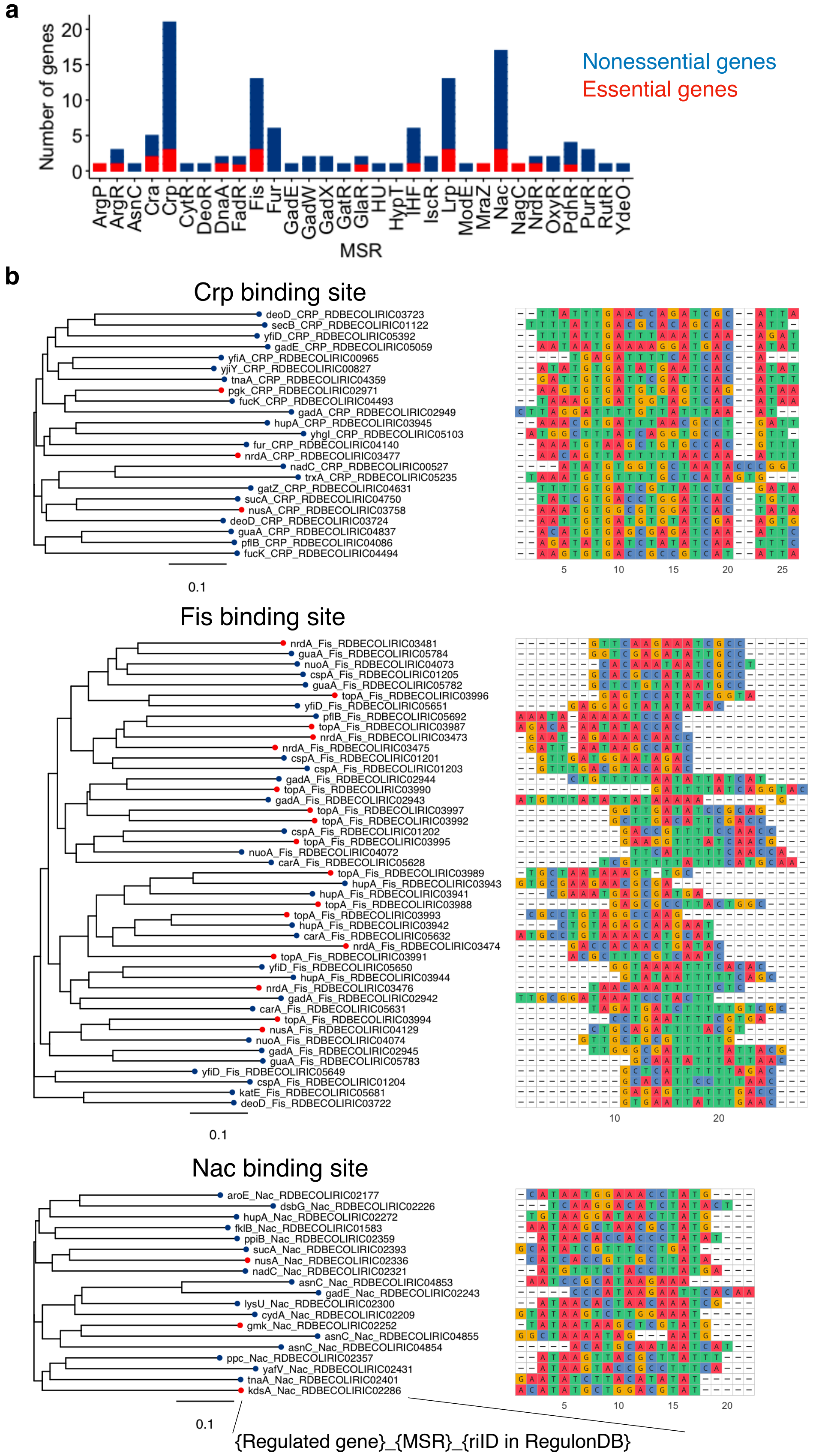

Fig. S3: Profiles of MSRs in essential and nonessential genes

a, The number of genes analyzed in this study for each metabolic sensory regulator (MSR). **b**,

Phylogenetic trees (left, constructed using the neighbor-joining method) of known binding site motifs for selected MSRs (Crp, Fis, or Nac) based on multiple sequence alignment (right). Binding site motifs were identified from RegulonDB[2]. The tip labels of the phylogenetic trees consist of gene name, MSR, and the regulatory interaction ID (riID) in RegulonDB. The tip colors represent gene essentiality.

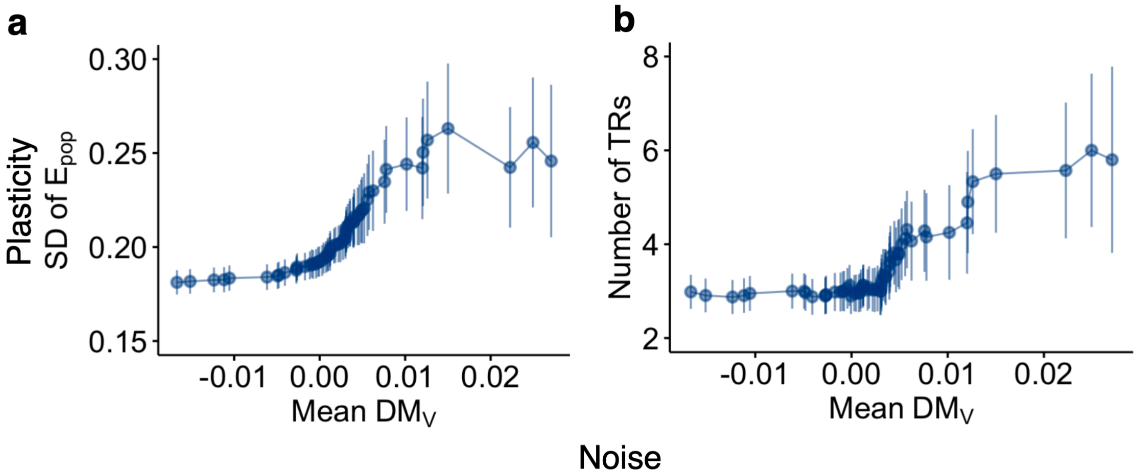

Fig. S4: A cumulative analysis for relationship between the number of transcriptional regulators, plasticity, and noise

Following a previous study (Figure 2 in Wolf *et al*[1]), a cumulative approach was employed to explore the relationship between the number of transcriptional regulators, plasticity, and noise in protein expression level. **a**, Genes were sorted by their noise level (x) As a function of a cut-off on x (horizontal axis), the mean and standard error (vertical axis) of plasticity in protein expression levels were calculated for all genes with the noise greater than x. **b**, Genes were sorted by their noise level x as in panel **a**, and the mean and standard error of the number of TRs including MSRs were calculated for all genes with noise level greater than x. The first cut-off (rightmost point) was set at the noise level of the sixth gene in the sorted list to ensure that each data point and error bar correspond to more than four genes for all panels. Means and standard errors are shown as the points and error bars. Nonessential genes were considered.

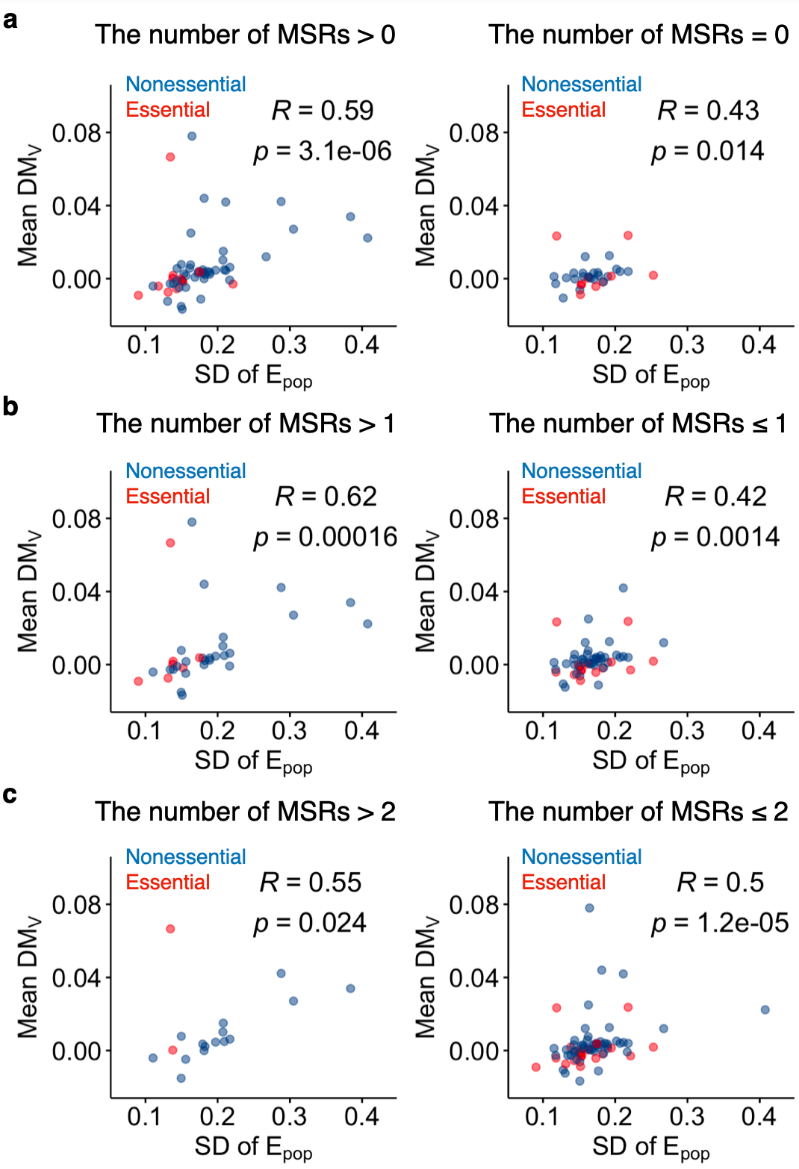

Fig. S5: Relationship between noise and plasticity for pooled genes

Genes are divided into two groups based on the number of MSRs. For each of the three cut-off levels (0 for **a**, 1 for **b**, and 2 for **c**), genes with more than the specified cut-off level of MSRs (left) and the remaining genes (right) were pooled separately, regardless of gene essentiality. Spearman's R and p-value are shown.

a

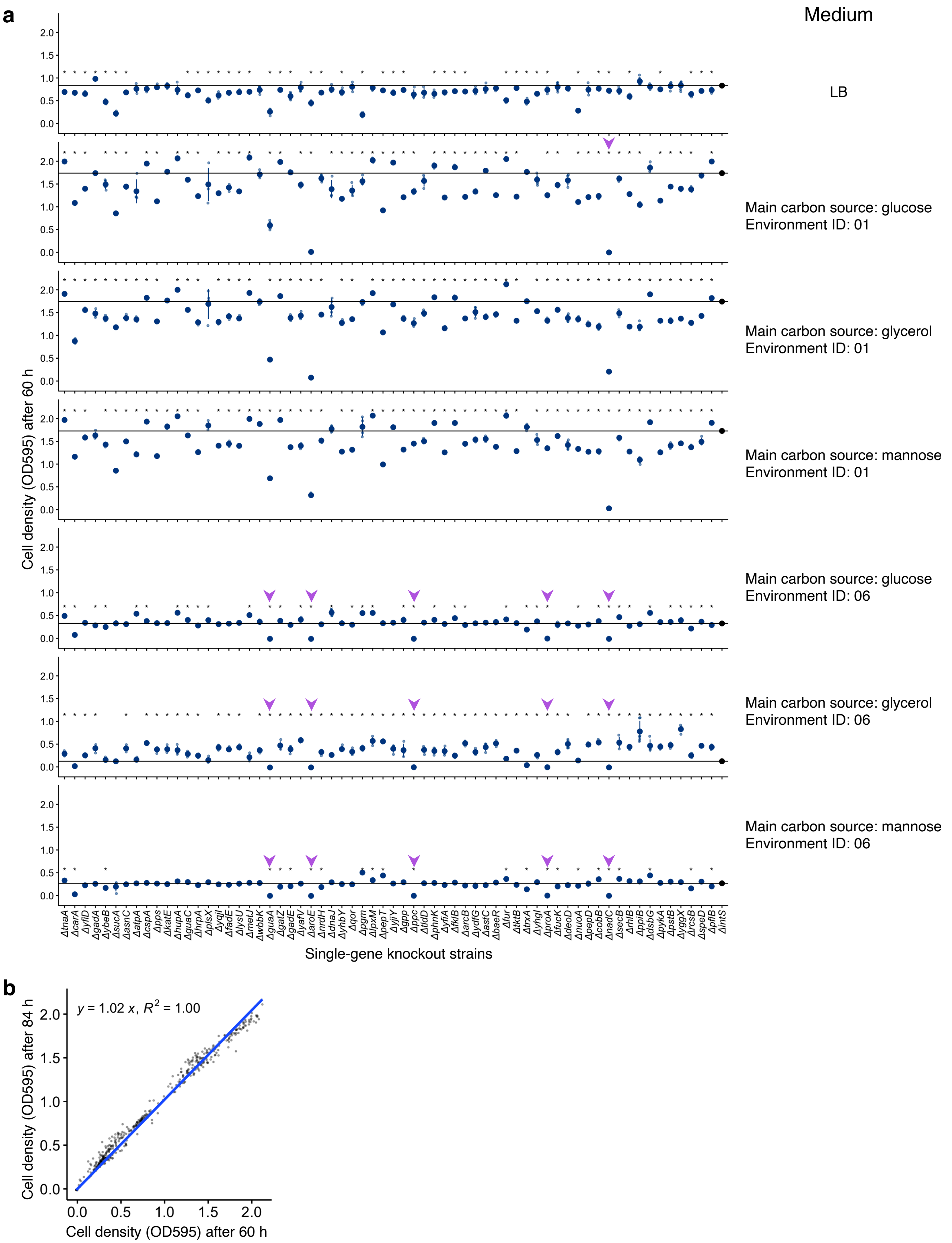

Fig. S6: Growth of single-gene knockout strains under different nutrient conditions

a, Single-gene knockout strains of 64 nonessential genes (**Supplementary Table S5**) and a control strain (*ΔintS*) were cultured under seven nutrient conditions. Growth yields were measured as cell

density (OD595) after 60 hours of incubation. The mean plus four standard deviations of OD595 in sterile cultures were subtracted. Small jittered points represent biological replicates (4–6 replicates per strain). Large circles and error bars indicate the mean and standard deviation among biological replicates. Horizontal lines denote the cell densities of the control strain (the rightmost strain in each panel). Asterisks indicate statistical significance (adjusted p-value<0.05, BH method) in pairwise comparisons (two-sided t-test) against the control strain. Arrowheads indicate no detectable growth, as detailed in **Methods**. **b**, Comparison of cell density between 60-hour and 84-hour incubation periods. Points represent the mean cell densities among biological replicates. The solid line represents a linear model fit without an intercept (y=1.02x), confirming confluent growth.

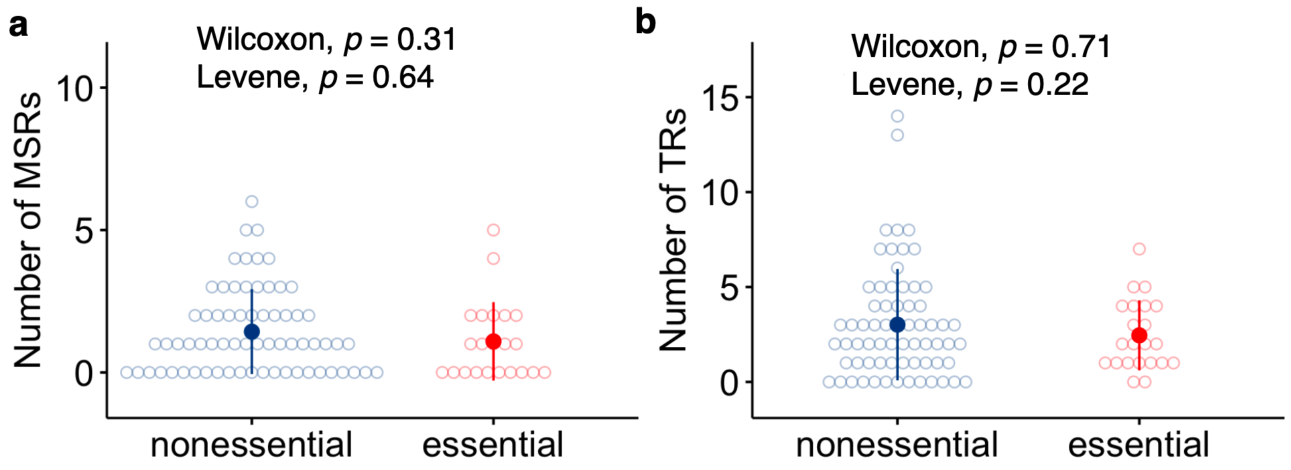

Fig. S7: Relationship between the number of transcriptional regulators and essentiality

a, Relationship between the number of MSRs and essentiality of target genes for cellular growth. **b**, Relationship between the number of all TRs, including MSRs, and essentiality of target genes. Means and standard deviations are shown as the large points and the error bars, respectively. Wilcoxon and Levene tests were examined for the mean and variance between two essentiality groups, respectively.

II. Supplementary References

1. Wolf, L., Silander, O. K. & van Nimwegen, E. Expression noise facilitates the evolution of gene regulation. *Elife*. **4** (2015).
2. Tierrafria, V. H. *et al.* RegulonDB 11.0: Comprehensive high-throughput datasets on transcriptional regulation in *Escherichia coli* K-12. *Microb Genom*. **8** (2022).
